# Traditional Male Circumcision in Uganda: A Qualitative Focus Group Discussion Analysis

**DOI:** 10.1371/journal.pone.0045316

**Published:** 2012-10-17

**Authors:** Amir Sabet Sarvestani, Leonard Bufumbo, James D. Geiger, Kathleen H. Sienko

**Affiliations:** 1 Design Science Program, University of Michigan, Ann Arbor, Michigan, United States of America; 2 Family Health International, Kampala, Uganda; 3 Department of Pediatric Surgery, University of Michigan, Ann Arbor, Michigan, United States of America; 4 Department of Mechanical Engineering, University of Michigan, Ann Arbor, Michigan, United States of America; 5 Department of Biomedical Engineering, University of Michigan, Ann Arbor, Michigan, United States of America; Yale School of Public Health, United States of America

## Abstract

**Background:**

The growing body of evidence attesting to the effectiveness of clinical male circumcision in the prevention of HIV/AIDS transmission is prompting the majority of sub-Saharan African governments to move towards the adoption of voluntary medical male circumcision (VMMC). Even though it is recommended to consider collaboration with traditional male circumcision (TMC) providers when planning for VMMC, there is limited knowledge available about the TMC landscape and traditional beliefs.

**Methodology and Main Findings:**

During 2010–11 over 25 focus group discussions (FGDs) were held with clan leaders, traditional cutters, and their assistants to understand the practice of TMC in four ethnic groups in Uganda. Cultural significance and cost were among the primary reasons cited for preferring TMC over VMMC. Ethnic groups in western Uganda circumcised boys at younger ages and encountered lower rates of TMC related adverse events compared to ethnic groups in eastern Uganda. Cutting styles and post-cut care also differed among the four groups. The use of a single razor blade per candidate instead of the traditional knife was identified as an important and recent change. Participants in the focus groups expressed interest in learning about methods to reduce adverse events.

**Conclusion:**

This work reaffirmed the strong cultural significance of TMC within Ugandan ethnic groups. Outcomes suggest that there is an opportunity to evaluate the involvement of local communities that still perform TMC in the national VMMC roll-out plan by devising safer, more effective procedures through innovative approaches.

## Introduction

HIV/AIDS remains a major health challenge throughout the world, especially in sub-Saharan Africa, where it accounts for 68% (or 22.5 million) of global HIV cases [1]. The use of male circumcision as an efficacious biomedical intervention against HIV transmission has been demonstrated in three randomized controlled clinical trials [2]–[4], which show a consistent protective effect of approximately 60% risk reduction among heterosexual men. More than 35 epidemiological studies [5]–[6] reinforce the results of the controlled trials. Faced with such evidence, the governments of most sub-Saharan countries are adopting policies and programs to “roll-out” voluntary medical male circumcision (VMMC) with the support of international public health organizations such as the World Health Organization and USAID [7]. In 2009, the Ugandan Ministry of Health (MoH) began to discuss a national plan for voluntary mass circumcision of adult males [8].

In many of these countries, traditional male circumcision (TMC) has been practiced for centuries, particularly as an initiation ritual and rite of passage into manhood [9]. As scale-up plans for clinical male circumcision are being considered as a strategy against HIV/AIDS by sub-Saharan African Ministries of Health, traditional providers will continue to function as an important source of service [10]. In fact, many international public health organizations believe that clinical male circumcision will never completely replace traditional practices due to both the cultural implications and the human resource constraints pending in the near future [9], [11]. Typically, providers with limited or no formal clinical training perform TMC in non-clinical settings. While some evidence supports TMC's effectiveness against HIV transmission [12]–[13], the life-threatening risks and health complications of its practice are alarming. Studies evaluating the complications due to TMC have found rates varying from 35% (Kenya) to 48% (South Africa) [5], [14]. Infection, delayed wound healing, glans amputation and injury, bleeding, loss of penile sensitivity, excessive removal of foreskin, and death are the major complications reported [5], [14]–[17].

Uganda's HIV prevalence rate is 6.5%, and almost 70% of Ugandan males remain uncircumcised [18]. Approximately 10% (3.5 million) of the population belongs to ethnic groups which still practice TMC [18]. The Ugandan National Safe Male Circumcision policy, a roadmap for implementation of an effective male circumcision program, acknowledges the importance of understanding TMC and its associated cultural aspects when devising methods to make TMC safer. Two suggested approaches, based on experiences in other countries, include the integration of TMC into official health care systems and the intensive training of traditional providers [5], [19]–[20]. Considering both the limitations of implementing VMMC in areas traditionally practicing circumcision and the promise of TMC for reducing infection transmission, the objective of this paper is to characterize TMC practices in Uganda and the cultural implications by using a comprehensive focus group discussion (FGD)–based qualitative analysis. Ultimately, such information can inform the strategies to make TMC safer and to fully utilize the resources available to support Uganda's gradual transition towards VMMC.

## Methods

To our best knowledge, this study is the first countrywide FGD-based qualitative analysis to understand the culture, traditions, and customs of TMC in Uganda.

### Ethics statement

The study was reviewed by the Institutional Review Board (IRB) of the University of Michigan in Ann Arbor, Michigan, USA, which determined that it met US federal criteria for exemption, including not more than minimal risk to subjects (exemption #2 (45 CFR 46.101(b)(2)). The University of Michigan's IRB informed the Uganda National Council of Science and Technology about this study and its exempt status. All study team members received training in the ethical conduct of human subjects' research. There were two data collection periods (2010 and 2011) utilizing focus groups. Although the study was considered exempt, participants were fully informed about the nature of the study prior to each FGD and were asked for their verbal consent. Also, they were able to leave at any time during the discussions; however, none of the participants opted to leave prior to the completion of the focus groups. Participants during the 2010 data collection sessions also provided written consent. For the focus groups conducted in 2011, the consent process was also audio recorded. No form of identifier (name, age, living location, clan) was collected from the participants. During FGDs, participants were assigned numbers or responded anonymously.

### Focus group discussion settings

**Figure 1:**
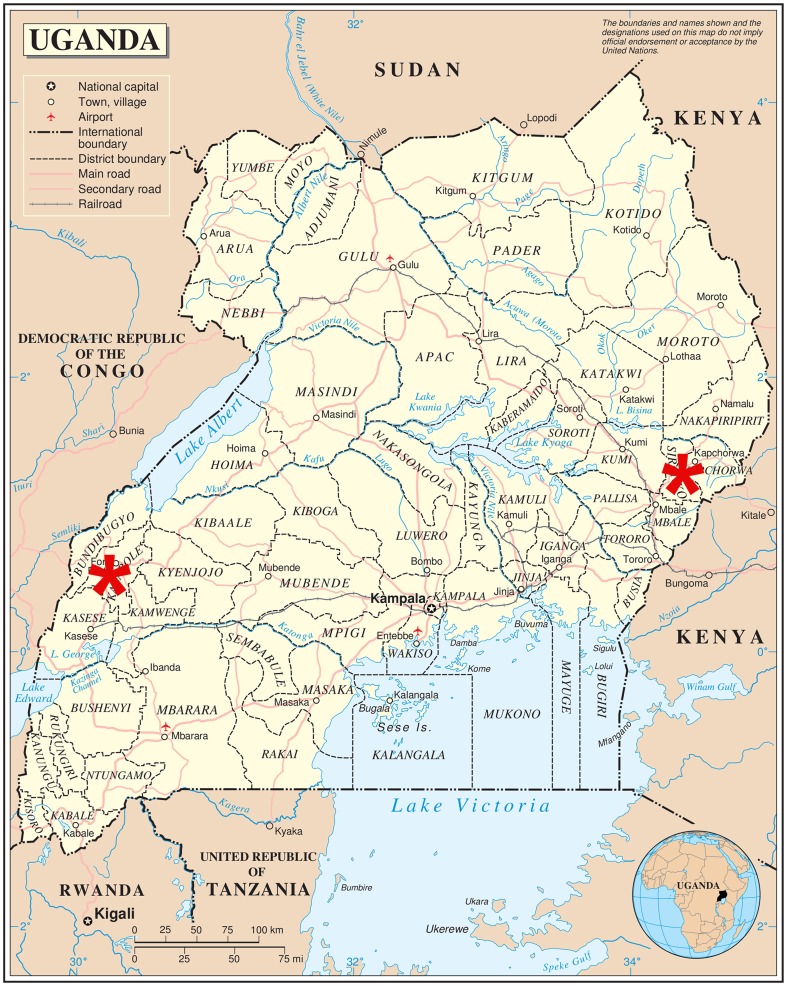
Map of Uganda. Stars indicate locations of FGDs. Source: Central Intelligence Agency World Factbook.

In Uganda, Sebei, Bagisu, Baamba, and Bakonzo ethnic groups practice TMC. The Sebei and Bagisu ethnic groups reside in eastern Uganda, while the Baamba and Bakonzo people reside in the western region. The HIV rate for Bagisu and Sebei men is 3.5%, while that of Baamba and Bakonzo men is 5.7% [18]. It is estimated that 80% of Sebei and Bagisu men are circumcised. The circumcision percentage of Baamba and Bakonzo men is unknown [18]. The study team held 26 FGDs (total of 208 participants) from August 2010 to June 2011. Each focus group consisted of 6–12 participants and was run by trained US and Uganda study team members, who remained the same across FGDs. Focus groups were held in local health clinics in Kapchorwa and Mbale districts (eastern Uganda) and Bundibugyo and Kasese districts (western Uganda), as indicated with red stars in Figure 1, and lasted for approximately 1 hour. They were conducted in the local language and translated simultaneously into English by an interpreter from the same ethnic group, who was trained in social science research and familiar with TMC. The participants were paid 10,000 Ugandan Shillings (UGX), or about USD 3.75, to reimburse their transportation expenses and time of participation.

### Participants

Three primary groups participated in the FGDs. Group one included traditional senior cutters responsible for cutting procedures. Group two included assistant cutters or guardians who help prepare boys (candidates) for circumcision, assist during the procedure, and advise candidates on post-operative care. Group three included clan leaders, who serve as community gatekeepers responsible for preserving the cultural aspects, such as TMC, of their respective ethnic groups. Each primary group attended a separate FGD designated by specific ethnicity. Table 1 shows the location, number of participants, and the groups' degree of involvement in the FGDs.

**Table 1:** Participant background and demographics.

Ethnic Group	FGD Location	Cutters	Assistant Cutters/Mentors	Clan Leaders	Total (%)
Sebei	Kapchorwa	20	21	22	**63 (30.3%)**
Bagisu	Mbale	14	16	16	**46 (22.1%)**
Baamba	Bundibugyo	11	10	17	**38 (18.3%)**
Bakonzo	Kasese	22	21	18	**61 (29.3%)**
**Total (%)**		**67 (32%)**	**68 (33%)**	**73 (35%)**	

### Focus group discussion topics

Focus groups were structured around the following topics:

1. Cultural and traditional significance of TMC.
2. General information on TMC.
3. Roles, responsibilities, and training processes for cutters and assistant cutters/guardians before, during, and after TMC.
4. Cutting techniques and handling of TMC adverse events.
5. Recent changes in TMC, and views and suggestion on how to make TMC safer.

### Qualitative data collection and management

Predetermined themes, such as TMC's cultural importance, logistics of the practice, cutters' training procedure, and tools used during TMC were selected prior to holding the FGDs. Several experts reviewed the planned themes and associated questions. The FGDs were audio and video recorded. All files were transcribed verbatim by two of the study team members. Study team members also cross checked the transcription results to ensure rigor and accuracy. Transcripts were reviewed, and reoccurring themes based on the five topics above were identified to develop a codebook. After an in-depth review of the transcriptions and cross-analyses of the four ethnic groups (Sebei, Bagisu, Baamba, Bakonzo) and different participant groups (clan leaders, traditional cutters, assistant cutters) additional codes were derived for further characterization. Hence, the codebook, which was initially based on predetermined codes, evolved through an iterative process with the emergence of new information, which was either unique to a given ethnic group or common across all groups.

## Results

### Cultural and traditional significance of TMC

In order to understand the cultural and traditional importance of TMC in each ethnic group, open-ended questions such as the following were asked:

1. What are the traditions, customs, and rituals associated with male circumcision in your ethnic group?
2. What are the reasons parents decide to circumcise their sons traditionally?

All participants agreed and even emphasized that traditional male circumcision is a major milestone in the process of becoming a man.

*“It [circumcision] is the time when a boy is initiated to become a man, to become his own person, when he has to take responsibilities. Traditionally, if a boy not cut traditionally will not be allowed to inherit and always will be called coward. Once he is born, family knows he must be cut traditionally. He is raised with that mentality and prepared for that important day [sic].”* (clan leader – Bagisu)

*“Once the boy is born, they know that he must be circumcised traditionally. So boys are brought up knowing they have to be circumcised in a traditional way [sic].”* (clan leader – Bagisu)

*“The process begins with dancing. The initiate goes around inviting his relatives and friends to attend the ceremony. Until the last day that is called the eve of the circumcision. That's when some rituals are done and in the morning the cutting is done [sic].”* (clan leader – Sebei)

The Bugisu region (eastern Uganda, Bagisu ethnic group) is considered the birthplace of TMC in Uganda. Common belief holds that the first male circumcision was performed in the region centuries ago. Even today at the start of each circumcision season, the first cohort of candidates is circumcised in the Bugisu region. This tradition is part of the cultural belief system to such an extent that those who are not circumcised traditionally are strongly stigmatized within their communities.

*“There is a big difference between a person circumcised at the hospital and one circumcised at home. Reason being that if you were circumcised in the hospital then you will never be an heir. And also if a child is going to be circumcised, you cannot advise because you did not go through a normal circumcision. When you are circumcised in the hospital, people look down upon you and know you are not as strong as others [sic].”* (clan leader – Bagisu)

In the Sebei and Bagisu ethnic groups, candidates announce their decision to be circumcised by dancing publicly in their villages a few days prior to the day of circumcision. They visit the homes of their relatives and invite them to the circumcision ceremony. During this time, they receive gifts from their relatives and help their parents prepare food and brew beer for the ceremony.

In the Baamba ethnic group, to ensure the safety of the procedure, sometimes a male relative of the candidate, typically a maternal uncle, stands behind the cutter, armed with a spear and ready to strike the cutter if the cut injures the boy in an unexpected way.

*“Family head stands behind the senior cutter holding the spear. The reason for it is that, if in any case, the procedure was done badly leading to death, then he would hit the cutter [sic].”* (clan leader – Baamba)

When asked if there were reasons for TMC beyond cultural beliefs, some participants from different ethnic groups cited health benefits.

### Candidate's age, TMC's season, cost, cutting time, and number of traditional cutters

Sample questions to stimulate discussion on the logistics and operations of TMC included the following:

1. What is the age range of the boys when they are circumcised?
2. What time of year is TMC performed?
3. How many circumcisions, on average, does each cutter perform during this time frame? How many traditional cutters are associated with your ethnic group?

Table 2 shows the candidates' age range, ethnic group, season, and the associated cost. The highest number of TMCs occurs in August and December due to school holidays. In eastern Uganda TMC is performed only in even years, while in western Uganda TMCs can be performed at any time depending on demand.

**Table 2:** General information on TMC for the four ethnic groups studied.

Ethnic Group	Age Range (yrs)	Circumcision Season	Cost Range	Cutting Time (sec)	Active Cutters
Sebei	14–18	Every even year, months of August and December	UGX 20,000–40,000 (USD 8–16)	10–50	20
Bagisu	14–18	Every even year, months of August–September and December–January	UGX 5,000–15,000 (USD 2.0–6.0)	5–10	1000
Baamba	5–15	Every year, months of August and December	UGX 5,000 (USD 2.0)	120–180	20
Bakonzo	2–15	Every year, months of August and December	UGX 5,000–15,000 (USD 2.0–6.0)	120–180	20

There is no fixed age limit in any of the ethnic groups, but the age range for eastern Ugandan candidates is relatively older (14–18 years) than that of western Uganda (2–15 years). The cost of TMC varies from UGX 5,000 to 40,000, or approximately USD 2.00 to 16.00 (Uganda GDP per capita is USD 1,300.00). The candidate's parents are responsible for the payment, although the price is negotiable and depends on the family's financial ability. Cutters performing procedures in the Sebei ethnic group are given a chicken and 20–40 liters of locally brewed beer in addition to the cash payment. Almost half of what a cutter receives must be given to his assistant.

When asked about the number of cutters in active practice, the Sebei, Baamba, and Bakonzo indicated about 20 cutters and the Bagisu indicated about 1000 cutters. This very high number is due to the Bagisu's growing population, the historical importance of TMC, and the social emphasis on training more cutters to meet demand. The average number of cuts performed by each cutter in each season is 170 (Sebei), 90 (Bagisu), and 200 (Baamba and Bakonzo). Cutting time is significantly shorter in the Bugisu and Sebei regions (Table 2).

### Role, responsibilities, and training process for cutters and assistant cutters/guardians, before, during, and after TMC

The following open-ended questions were asked to learn about the role of senior and assistant cutters and to understand whether they underwent any systematic training:

1. Can you describe your role (as a cutter/assistant cutter) during the traditional circumcision in detail?
2. What do you do to prepare the candidate before and after TMC?
3. What makes one cutter better than another?
4. What type of training, if any, is required to become a cutter or assistant cutter/mentor?

The Sebei did not have a traditional cutter of their own until the mid-1980s; instead they asked Bagisu cutters to perform the procedure. However, in the last 20 years, the Sebei trained their cutters by shadowing those of the Bagisu group.

*“We thought of the money they [Bagisu cutters] were making. We thought why are we losing this money? That is why we started performing circumcision [sic].”* (clan leader – Sebei)

A Sebei cutter's role is simply to perform the actual cut of the foreskin.

*“A good cutter is the one who cuts fast, but does not hurt the head of the penis.” “[a good cutter is determined] based on the size of the wound. The quicker it heals means the person who circumcised is better in cutting.” “A good cutter is one who cuts and no [foreskin] part is left. So, during the healing process the mentors have been able to identify these cutters and let the community know [sic].”* (cutter – Sebei)

Most Sebei cutters lack formal training, other than occasional meetings with others involved in TMC to talk about their experiences, and shadowing elders.

*“In some cases they [cutters] have seminars among themselves that's coordinated by their seniors, those who have been cutting for a long time and have been training them [sic].”* (cutter – Sebei)

Sebei cutters who attended the FGDs had been practicing on average for 10.5 years. Assistant cutters in Sebei are referred to as “guardians or mentors” and are responsible for coaching the candidate, preparing him for the cut, and advising him on post-operative care for the wound. Guardians also ensure that a clean knife is used for each candidate and that cutters wash their hands before the procedure.

*“Mentors assist cutters to make sure that candidates have been circumcised very well [sic].”* (clan leader – Sebei)

A good mentor is one whose candidates do not fear the procedure and whose recovery periods are one week or less. On average, Sebei guardians who participated in the FGDs had 14 years of experience.

Notably, only the Bagisu group has formed a union of cutters and assistant cutters and registered the organization with the local government. Not everyone within the Bagisu group can become a cutter, since the journey is a spiritual one that is not afforded to many. The process typically starts with the onset of a mysterious sickness, during which the individual dreams of ancestral spirits which encourage him to become involved in TMC. When the individual falls ill and does not respond to traditional or modern medicine, he is taken to the elders of the community. Depending on the situation and the individual's background and circumstances, the elders decide if he is ready to become involved in TMC. If accepted by the elders, the individual begins to shadow a senior cutter as an assistant.

A few days before each circumcision season, the local district health office in the Mbale District holds training sessions for TMC cutters and their assistants that provide instruction on safe and hygienic practices and adverse events management. Cutters must obtain a certificate from the district health office upon finishing the training session before they can perform that season. Cutters in the Bagisu group are solely responsible for the circumcision cut and the assistant cutters are responsible for preparing the candidate. A good Bagisu cutter should hold strong ties to the community and know how to make a fast cut without complications. Senior cutters attending the FGDs had been working as senior cutters on average for 13 years.

Assistant cutters take instructions from senior cutters. The assistants manage and control the crowds, which typically gather at the circumcision ceremony, ensuring that the cutter and candidates are not disturbed. They also care for the wound following the procedure. The Bagisu group requires its assistants to shadow senior cutters extensively before the seniors and clan leaders determine whether they are ready to graduate to senior cutter. Bagisu assistant cutters who participated in the FGDs had been working as assistant cutters on average for 11 years.

Among the Baamba and Bakonzo, TMC is considered a family business. Cutters and assistant cutters from both ethnic groups who participated in the FGDs said they were involved in TMC because of their fathers and grandfathers. No formal training exists in either ethnic group. Rather, a good cutter typically performs a consistent cut, leaves a minimal amount of foreskin, and uses a new razor blade for each candidate.

*“In order for somebody to become a senior cutter, it is about consistency and speed in the [cutting] procedure [sic].”* (cutter – Baamba)

A senior cutter must learn to effectively manage complications. To prevent possible complications, Bakonzo cutters frequently visit candidates post-procedure to clean the wounds and advise parents on proper care. In the Baamba and Bakonzo groups, cutters who participated in the FGDs had been working on average for 40 and 24 years, respectively.

Assistant cutters in both ethnic groups hold young candidates on their laps while the cutter performs the circumcision. In the Baamba ethnic group, assistant cutters remain with the candidate for a few hours post-procedure to care for the wound and manage potential complications. A Baamba assistant cutter explained:

*“We wash the wound after cut with water. We also stay around for few hours to take care of the boy to make sure he is fine. Then, we hand him to his parents [sic].”* (assistant cutter – Baamba)

Assistant cutters in the Bakonzo remain with the candidate for a half hour post-procedure. Assistant cutters in the Baamba and Bakonzo who participated in the FGDs had been working on average for 10 and 22 years, respectively.

### Cutting techniques and handling of TMC adverse events

To obtain information about cutting techniques unique to each ethnic group, their associated adverse events, and the view of local communities on potential changes to make TMC safer, the following questions were asked:

1. What are the techniques used for traditional circumcision cuts in your ethnic group? Is there any variation among cutters' methods? How much foreskin is cut?
2. Have you ever heard of a circumcision that has resulted in an adverse event? If yes, what was the reason? Who is to blame if an adverse event happens?

While it should be acknowledged that there is no set TMC “style”, the majority of cutters in the Sebei and Bagisu groups share the same method. That is, a candidate ready to be circumcised is called to the center of the area designated for the circumcision ceremony. The boy stands and holds his hands up as the cutter removes his clothing to expose the penile shaft. The cutter pushes the glans inside and pulls the foreskin forward. The pushing and pulling sequence is performed three to four times While pulling the foreskin, he places his thumbnail where he can feel the glans. He uses his nail to mark where the glans ends and to protect it against the cut. While the foreskin is pulled, the cutter uses a traditional knife to cut through it. After the first cut, the assistant cutter holds the glans as the cutter removes the remaining foreskin (inner layer) through a radial cut using the same knife. Cutters do not dress the wound with any medical supplies. Clan leaders attending the ceremony are responsible for supervising the process.

*“Cutting method depends on the length of the foreskin. During the cutting ceremony clan leaders stand by the candidate and advise if there is too much or less skin cut. They also make sure the cutter acts responsibly if a complication happens [sic].”* (cutter – Sebei)

The major difference between Sebei and Bagisu cutting styles is that the Sebei do not cut some of the skin from the inner layer whereas the Bagisu cut the entire foreskin.

*“In compare to Bagisu, Sebei cut less amount of foreskin because cutting too much makes healing process complicated [sic].”* (cutter – Sebei)

**Figure 2:**
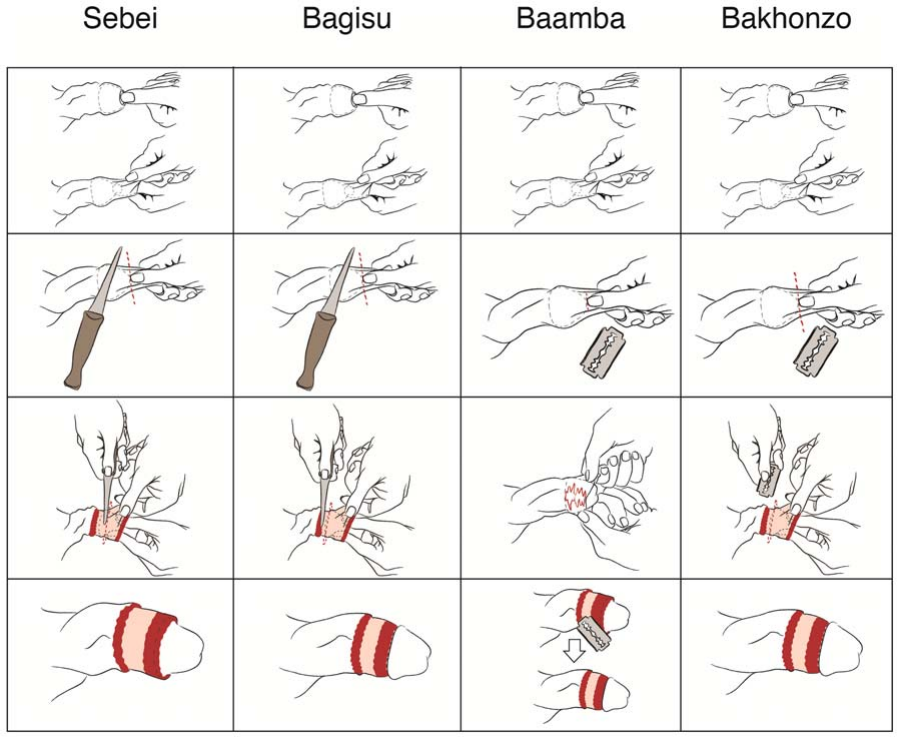
Illustration of traditional circumcision cutting techniques by ethnic group. Columns depict TMC cutting techniques per ethnic group. Rows show cutting process steps (row 1: pull foreskin and push glans; row 2: initial cut; row 3: secondary cut; row 4: circumcised penis).

Figure 2 and Table 3 summarize the cutting techniques used by the four ethnic groups. As shown for the Sebei and Bagisu, the first two cutting steps are identical. But for the second cut, Sebei cutters leave some foreskin intact. The final row of images shows the outcome of the traditional cut. The pink area shown is a layer of inner foreskin. The red area depicts the open wound caused by the cut.

**Table 3:** Circumcision cut style and performer per ethnic group.

Ethnic Group	Cut Performed by	Cutting Style
Sebei	Cutter	Push the glans in. 2. Pull the foreskin forward. 3. Cut through foreskin with a traditional knife. 4. Hold the glans and perform a radial cut. Leave some amount of foreskin uncut.
Bagisu	Cutter	Push the glans in. 2. Pull the foreskin forward. 3. Cut through foreskin with a traditional knife. 4. Hold the glans and perform a radial cut. Remove the foreskin fully.
Baamba	Cutter with assistant cutter	Push the glans in. 2. Pull the foreskin forward. 3. Make an incision through foreskin with a razor blade. 4. Tear apart the foreskin by hand. 5. Cut any remaining foreskin through a radial cut with a razor blade.
Bakonzo	Cutter	Push the glans in. 2. Pull the foreskin forward. 3. Cut through the foreskin with a razor blade. 4. If the cutter feels the inner layer is long, perform a radial cut.

In the Baamba ethnic group, a candidate arrives at the designated cutting area and the cutter strips him down. If too young to stand alone, the boy is held by a male family relative or by the assistant cutter. After exposing the penile shaft, the cutter pulls the foreskin to measure the amount to be cut. Similar to the process followed by the Bagisu and Sebei, the cutter uses his thumbnail to indicate where the cut should be made. A razor blade provided by the parents of the candidate is used to make a small incision to allow the cutter and his assistant to tear apart the skin. Once the incision is made, the assistant cutter tears the skin by pulling it apart up to the penis corona. Finally, the cutter uses the razor blade to cut away any remaining skin (Fig. 2). After the cut, the assistant cutter washes the penis with clean water, but does not use medical supplies to dress the wound. Cutters in Bakonzo explained their method as a simple pull on the foreskin followed by a cut through it with a razor blade (Fig. 2). If they feel the inner layer is too long, they cut it radially around the penile shaft, otherwise the first cut suffices. In this technique, the cutting style depends on candidate's age. If the boy is younger than five years old, the cutters usually perform an initial cut and a radial cut. If the candidate is older, one vertical cut is enough to consider the boy circumcised.

Participants in all of the FGDs identified excessive bleeding, prolonged wound healing, infection, glans injury and amputation, and unfinished cuts requiring additional cuts as the most common adverse events. Sebei and Bagisu participants also mentioned the risk of deafness due to excessive festivities with loud music and crowds.

*“Complications happen due to rushing and the speed of the process. There will be inaccuracy and imperfect cutting by the cutter [sic].”* (clan leader – Sebei)

One Bagisu cutter complained about the uncontrollable and crowded public who surround the candidate and cutter to watch the ceremony:

*“Sometimes the complications they [candidates] are getting is because of the rowdy crowd. Sometimes they become so crowded and they push you [sic].”* (cutter – Bagisu)

No focus group participant would identify the party responsible for an adverse event, although a few cutters blamed their assistant or the candidate, citing the failure to adequately care for the wound. Assistant cutters and clan leaders mostly blamed the cutters, claiming that it was their responsibility to ensure the candidate's safety.

### Recent changes in TMC, views, and suggestions for making it safer

To capture recent changes to the traditional circumcision ceremony and to explore the potential for additional future changes to make TMC safer, the following questions were asked:

1. Have the traditions, customs, and rituals associated with circumcision in this region changed over time? If yes, how? Why?
2. Would you support changes in TMC practice to make it safer? What type of changes would you considering?

As mentioned, custom, ritual and cutting methods vary by ethnic group. However, the use of one traditional knife or razor blade per candidate during circumcision is one of the most significant changes mandated by the Uganda MoH. The change was implemented in early 2000 across all ethnic groups. Eastern groups still use a traditional knife whereas the Baamba and Bakonzo groups use razor blades.

*“Due to country's development of change of time, now we have changed some customs and rituals. Now, we use one-time use razor blades and have made the cutting procedure and ceremonies more decent [sic].”* (cutter – Baamba)

*“Cutters nowadays must have different [separate] knives per candidate [sic].”* (clan leader – Bagisu)

Another change is connected to the spread of organized religions in Uganda. For instance, Muslims prefer to circumcise their sons at an early age (typically 7 days old). Catholics and Anglicans oppose the excessive festivities surrounding TMC, the over-consumption of alcohol, and promiscuity. Hence, an ethnic group's religious preference can motivate a change in TMC practice.

*“For some people, due to their modern religious beliefs, they don't participate in dancing ceremonies. They just cut traditionally and leave it at that [sic].”* (clan leader – Bagisu)

*“Initially we were using traditional knives, just very sharp and small. There is now a razor blade per candidate. Each candidate is also provided with his own water. After circumcising him, we wash the fresh cut with clean water [sic]”* (cutter – Baamba)

Although there have been changes in custom and rituals, a Bagisu cutter expressed:

*“No matter what has changed around circumcision, the bottom line and the most important factor is that the boy must be cut traditionally [sic].”* (cutter – Bagisu)

Participants were also asked about potential reforms in TMC that can help reduce its adverse events.

*“We accept promoting other tools for circumcision. When we are looking at how the world has been in the past and now, there have been many complications [with TMC], so we are positive to adopt scissors and razor blades for the procedures, as long as it reduces the risks to the circumcision [sic].”* (clan leader – Sebei)

*“In villages lack the equipments, so if there is a way, a tool, that specifically can reduce the pain and maybe fast healing, we can welcome it very well [sic].”* (clan leader – Bakonzo)

The majority of the FGDs emphasized that information on the importance and health benefits of circumcision should be provided and that families should be informed about what to look for when selecting a cutter.

*“Better is that to make people educated to know how to have circumcision safe. Unless we educate them about that complications will continue [sic].”* (cutter – Bagisu)

Most participants also stressed the need to inform people about adverse events. When asked about venues to disseminate such information and by whom, the participants cited: churches and mosques (religious leaders); radio talk shows (clan leaders); and schools (teachers). Other suggestions included stocking health clinics with wound-dressing supplies, clean gloves, and sterile razor blades for cutters to purchase for a minimal fee.

## Discussion

In Uganda, as in most other sub-Saharan African countries where TMC is practiced, traditional circumcision marks the entry to manhood. However, there are variations in the logistics and performance of TMC among Uganda's four ethnic groups. For instance, eastern groups tend to circumcise at an older age than those in western Uganda. There are also variations in cutting styles. For example, even though Sebei cutters are trained by their Bagisu counterparts, they leave some of the foreskin intact unlike the Bagisu, who cut the entire foreskin. The side effects of such cutting style variations include longer healing times and potentially different protection levels against HIV/AIDS transmission; a report from the Forum for Collaborative HIV Research recommends leaving less than 3 mm of foreskin (although this is an on-going area of research) in a clinical circumcision for the most effective protection [21]. Complications cited by several focus group participants are consistent with the adverse events identified in previous studies [9]. They revealed that rapid cutting methods are effective in reducing instant pain but can increase the risk of glans injury and amputation and cause larger wounds and scarring. Participants from the Baamba and Bakonzo ethnic groups recalled fewer adverse events, which we attribute to the younger age of their candidates, the fact that Baamba assistant cutters remain with the patient for a few hours post-cut, and that Bakonzo cutters perform a follow-up visit a few days later.

For the Bagisu group, TMC represents a sense of pride. Unlike the three other groups, the Bagisu had formed a union comprised of cutters and assistant cutters to determine how to best preserve TMC's cultural significance in an era when festivities, elaborate dances, and other forms of celebration centered around TMC have been greatly reduced. In western Uganda, celebrations are rare and in the east they are shorter and less well attended. Focus group participants from various backgrounds emphasized that they would not completely abandon TMC, even if the side-events that typically accompany the ritual disappear. From a policy perspective, local communities' willingness to detach from some traditions signals a potential opportunity to discuss how to make TMC safer, but only if the local leaders are included in the planning and implementation.

Focus group participants offered several reasons for preferring TMC over clinical circumcision, such as cultural significance, low cost, and individual's resistance to the modern health care system. Although some participants were aware of the positive impact of circumcision in reducing HIV transmission, it is unclear whether traditionally circumcised males will experience the same level of protection from HIV transmission [12]–[13].

We suggest that a reliable clinical infrastructure providing voluntary mass medical male circumcisions by trained individuals using appropriate equipment is the best long-term solution to reduce circumcision-based HIV transmission rates in sub-Saharan Africa. However, a number of significant barriers identified by the African Ministries of Health and emphasized in our paper make it unlikely that the VMMC vision for Uganda will be realized in the near future [22]. Among the critical issues cited for the slow scale-up of clinical male circumcision are a shortage of human resources for programming and service delivery; a lack of buy-in from social gatekeepers such as traditional clan leaders and key decision leaders; and a poor understanding of how policy-makers might engage Ugandans in order to influence behavioral change [22]. The strong cultural significance of TMC reaffirmed through the FGDs demonstrates the reluctance of local communities to partake in the government's mass VMMC roll-out plan. However, timely changes in TMC practices, such as minimizing the TMC related festivities, using one knife/razor blade per candidate, and acceptance of local health staff supervision in some cases in Bagisu (e.g., mandatory training certificates by local health office for all cutters) demonstrate the possibility of acceptance in the future. Indeed, changing attitudes at the community level may open the door for health care providers, key decision-leaders, and policy-makers to explore a hybrid model that standardizes cutting style and ensures effective protection against HIV/AIDS transmission. Sharing responsibility between the trained health care provider who is responsible for the cut and caring of the wound and the local cutter who is responsible for cultural rituals might also mitigate the risks of excessive bleeding and glans damage, and reduce overall healing time.

The limited information about the effectiveness of TMC against HIV/AIDS suggests the need for both a systematic evaluation of TMC's role in HIV prevention and the creation of innovative approaches to reduce adverse events. While the initial attempt should focus on making TMC safer, communities that still practice TMC need to be made aware of VMMC's health benefits. For men who are already circumcised traditionally, the educational campaigns should provide information about the limited protective effects of TMC against HIV/AIDS to adjust for risk compensation behavior. The results of the FGDs support these and earlier suggestions to engage local communities that perform TMC in the planning and execution of an effective, safe mass male circumcision roll-out plan [22]. A meeting of NGO representatives and sub-Saharan African Ministries of Health officials who met in 2009 to discuss their progress with the mass scale-up of VMMC and to evaluate the common challenges, states that “it is important to maintain engagement with traditional circumcisers and to avoid alienating them and to use this opportunity for promoting safer traditional practices.” [22]. This is especially true in communities where TMC provides status and a source of revenue. Traditional cutters can be involved by educating them about sterile, hygienic practices and methods to manage complications and risks. The FGD results also demonstrate an opportunity for gradual transition of TMC practicing communities to accept VMMC. To implement such transitions and innovative approaches, collaboration can be undertaken with local religious and community leaders, and information about the importance of VMMC and the methods to reduce adverse events of TMC can be disseminated to Uganda's media, schools, and public venues.

This paper represents the first attempt to demonstrate the landscape of TMC in Uganda. However, the findings reported here should be considered with specific limitations. While the study team made great efforts to include a wide range of informed stakeholders, it is possible that the final study does not reflect the full spectrum of beliefs and opinions about TMC in Uganda. Nevertheless, considering the number of participants from different ethnic groups and the quality of the data collected, saturation was achieved and no new information emerged during the final FGDs. Opinions presented in the FGDs represent the knowledge, assumptions, and understanding of the participants. While the participants are considered experts in this field, their opinions may not reflect the most accurate facts about TMC. Furthermore, there may be minor grammatical (real-time translations reported herein without modification) and contextual related issues associated with translating the FGD participants' responses from their local languages to English. Finally, this work on four ethnic groups that practice TMC in Uganda may not be relevant for other communities in sub-Saharan Africa that also practice TMC. We conclude, however, that, communities' attitudes and reactions to change, common adverse events, and the challenges associated with making TMC safer are expandable concepts.

We suggest that our research is an important factor in developing both a safe TMC program and the educational and informing methods required for an effective national mass male circumcision roll-out. Further studies should be undertaken to evaluate the adverse events of TMC in Uganda and its potential effectiveness for public health purposes, and to identify the potential methods and approaches needed to convince local communities to adopt safe practices and potentially transition to VMMC.

## References

[1] UNAIDS (2010) UNAIDS report on the global AIDS epidemic 2010.

[2] Auvert B, Taljaard D, Lagarde E, Sobngwi-Tambekou J, Sitta R, et al (2005) Randomized, controlled intervention trial of male circumcision for reduction of HIV infection risk: The ANRS 1265 trial. PLoS Medicine 2 (11) 1112–1122. 10.1371/journal.pmed.0020298 PMC1262556 16231970

[3] Gray RH, Kigozi G, Serwadda D, Makumbi F, Watya S, et al (2007) Male circumcision for HIV prevention in men in Rakai, Uganda: a randomised trial. Lancet 369 (9562) 657–666. 17321311 10.1016/S0140-6736(07)60313-4

[4] Bailey RC, Moses S, Parker CB, Agot K, Maclean I, et al (2007) Male circumcision for HIV prevention in young men in Kisumu, Kenya: a randomised controlled trial. Lancet 369 (9562) 643–656. 17321310 10.1016/S0140-6736(07)60312-2

[5] Bailey RC, Egesah O, Rosenberg S (2008) Male circumcision for HIV prevention: a prospective study of complications in clinical and traditional settings in Bungoma, Kenya. Bull World Health Organ 86 (9) 669–77. 18797642 10.2471/BLT.08.051482 PMC2649497

[6] Herman-Roloff A, Llewellyn E, Obiero W, Agot K, Ndinya-Achola J, et al (2011) Implementing Voluntary Medical Male Circumcision for HIV Prevention in Nyanza Province, Kenya: Lessons Learned during the First Year. PLoS One 6 (4) 10.1371/journal.pone.0018299 PMC3070734 21483697

[7] World Health Organization and Joint United Nations Programme on HIV/AIDS (2008) Operational guidance for scaling up male circumcision services for HIV prevention. WHO Press.

[8] Uganda Ministry of Health (2010) Mass Male Circumcision Roll Out Plan. Uganda Ministry of Health.

[9] Wilcken A, Keil T, Dick B (2010) Traditional male circumcision in eastern and southern Africa: a systematic review of prevalence and complications. Bull World Health Organ 88 (12) 907–14. 21124715 10.2471/BLT.09.072975 PMC2995181

[10] Brown JE, Micheni KD, Grant EMJ, Mwenda JM, Muthiri FM (2001) Varieties of male circumcision: A study from Kenya. Sexually Transmitted Diseases 28 (10) 608–612. 11689759 10.1097/00007435-200110000-00007

[11] Wambura M, Mwanga JR, Mosha JF, Mshana G, Mosha F, et al (2011) Acceptability of medical male circumcision in the traditionally circumcising communities in Northern Tanzania. BMC Public Health 11: 373. 21605433 10.1186/1471-2458-11-373 PMC3112418

[12] Maughan-Brown B, Venkataramani AS, Nattrass N, Seekings J, Whiteside AW (2011) A cut above the rest: Traditional male circumcision and HIV risk among xhosa men in Cape Town, South Africa. Journal of Acquired Immune Deficiency Syndromes 58 (5) 499–505. 21909030 10.1097/QAI.0b013e31823584c1

[13] Shaffer DN, Bautista CT, Sateren WB, Sawe FK, Kiplangat SC, et al (2007) The protective effect of circumcision on HIV incidence in rural low-risk men circumcised predominantly by traditional circumcisers in Kenya: Two-year follow-up of the Kericho HIV Cohort Study. Journal of Acquired Immune Deficiency Syndromes 45 (4) 371–379. 17558336 10.1097/QAI.0b013e318095a3da

[14] Lagarde E, Dirk T, Puren A, Reathe RT, Bertran A (2003) Acceptability of male circumcision as a tool for preventing HIV infection in a highly infected community in South Africa. AIDS 17 (1) 89–95. 12478073 10.1097/00002030-200301030-00012

[15] Crowley IP, Kesner KM (1990) Ritual circumcision (Umkhwetha) amongst the Xhosa of the Ciskei. British Journal of Urology 66 (3) 318–321. 2207550 10.1111/j.1464-410x.1990.tb14936.x

[16] Magoha GA (1999) Circumcision in various Nigerian and Kenyan hospitals. East African Medical Journal 76 (10) 583–586. 10734511

[17] Meissner O, Buso DL (2007) Traditional male circumcision in the Eastern Cape - Scourge or blessing? South African Medical Journal 97 (5) 371–373. 17599221

[18] Uganda Ministry of Health (2006) HIV/AIDS sero-behavioural survey. Uganda Ministry of Health.

[19] Peltzer K, Nqeketo A, Petros G, Kanta X (2008) Traditional circumcision during manhood initiation rituals in the Eastern Cape, South Africa: A pre-post intervention evaluation. BMC Public Health 8: 64. 18284673 10.1186/1471-2458-8-64 PMC2259337

[20] Peltzer K, Kanta X, Banyini M (2010) Evaluation of a safer male circumcision training programme for ndebele traditional surgeons and nurses in Gauteng, South Africa: Using direct observation of circumcision procedures. African Journal of Traditional, Complementary and Alternative Medicines 7 (2) 153–159. 10.4314/ajtcam.v7i2.50876 PMC3021160 21304627

[21] Bakare N, Miller V (2008) Meeting the demand for male circumcision - Report of a workshopt convened by the forum for collaborative HIV research (Kampala, Uganda). World Health Organization.

[22] World Health Organization (2009) Country experience in the scale-up of male circumcision in eastern and southern africa region: two years and counting. Meeting report, Windhoek, Namibia.

